# Supplementary material for: MetaRibo-Seq measures translation in microbiomes
Source: Nat Commun. 2020 Jun 29;11:3268. doi: 10.1038/s41467-020-17081-z (PMC7324362; doi:10.1038/s41467-020-17081-z)
Supplement: Supplementary file 10 — Supplementary Data 7 [file 41467_2020_17081_MOESM10_ESM.zip › File2/Confidence_High_Taxonomy/97067_out.krona.html]

Javascript must be enabled to view this page.

members
magnitude
magnitudeUnassigned
count
unassigned
taxon
rank

97067\_out

2

superkingdom
2
2

phylum
1239
2

2
186801
class

order
186802
2

2
541000
family

2
genus
216851

2

SRS077024\_contig\_number\_14551SRS1041137\_contig\_number\_33396
species
853
